# Efficacy of Cabbage Leaf versus Cooling Gel Pad or Diclofenac Gel for Patients with Knee Osteoarthritis: A Randomized Open-Labeled Controlled Clinical Trial

**DOI:** 10.1155/2022/3122153

**Published:** 2022-06-08

**Authors:** Thanapon Chobpenthai, Patcharapol Arunwatthanangkul, Wiriya Mahikul

**Affiliations:** Princess Srisavangavadhana College of Medicine, Chulabhorn Royal Academy, Bangkok, Thailand

## Abstract

**Background:**

Osteoarthritis (OA) is one of the most common joint degeneration ailments adversely affecting the elderly population by impairing their physical movements and quality of life. This study aimed to establish the efficacy of cabbage leaf application in alleviating pain-related distress and positively improving OA conditions.

**Materials and Methods:**

Patients with moderate to severe (grades 3-4) OA by the Kellgren and Lawrence grading system with a poor to good Oxford Knee Score were selected for enrollment in this clinical trial. The participants were divided into three intervention groups: the cooling gel pad group for 20 minutes duration once a day (*n* = 20), the diclofenac gel group for 4 times a day (*n* = 20) as the control group (total *n* = 40), and the cabbage leaf group for 1-hour duration once a day (*n* = 20) as the experimental group (total *n* = 20). All trial participants were trained to record their Numerical Rating Scale (NRS) pain score and Oxford Knee Score and were advised to undergo weekly follow-ups and assessment of the outcome at 4 weeks. Data were analyzed by the paired *t*-test and analysis of variance (ANOVA).

**Results:**

The cabbage leaf group and cooling gel pad group showed a significant difference in both the Oxford Knee Score (*p* < 0.001 in both groups) and NRS score (*p* < 0.001 in both groups) before and after the intervention, by using the paired *t*-test. The three study procedures were found to be significantly different with respect to both the Oxford Knee Score (*p*=0.012) and NRS score (*p* < 0.001), by using ANOVA.

**Conclusion:**

This study clinically demonstrated that cabbage leaf application and cooling gel pad application showed similar improvements in reducing OA symptoms in terms of the overall NRS score and Oxford Knee Score. Their therapeutic effectiveness was better than that of diclofenac gel.

## 1. Introduction

Osteoarthritis (OA) is regarded as one of the most commonly occurring joint degeneration ailments in the older population aged >55 years [1]. The most common symptoms are pain and gait dysfunction [2–4], which significantly impair physical movements and substantially affect the quality of life [4–6]. Alternative nonoperative treatments for OA include physical therapy, medication, bandages, and applying compresses [1, 2, 4]. Guccione et al. [6] reported the use of cabbage leaf compresses to relieve pain in patients with OA. Lauche et al. [7] carried out a randomized controlled study to evaluate the efficacy of cabbage leaf therapy in patients with knee OA and observed a positive impact on the alleviation of OA symptoms with a reduction in pain, lessened functional disability after 4 weeks, and improvement in overall quality of life after 12 weeks. Clinical trials including those conducted by Boi et al. [8], Nikodem et al. [9], and Roberts et al. [10] demonstrated the efficacy of cabbage leaf compression in relieving the pain associated with breast congestion in breastfeeding mothers. A randomized controlled study of the effect of cold cabbage leaf compresses revealed a significant reduction in hardness and significant pain relief in patients with breast engorgement [11]. Furthermore, a quasi-experimental study conducted by Kaur and Saini [12] showed similar results among postnatal mothers treated by chilled cabbage leaf application and demonstrated it to be an effective home remedy in reducing the severity of breast engorgement. However, very few studies have focused on cabbage leaf compression for pain relief in patients with OA [7].

The main objective of this study was to establish the efficacy of cabbage leaf application in alleviating pain and positively improving OA conditions as an alternative nonoperative treatment compared with the application of a cooling gel pad alone and diclofenac gel application alone.

## 2. Materials and Methods

### 2.1. Trial Design

This randomized, controlled, three-arm parallel clinical trial involved patients treated at the Orthopedic Surgery Clinic of Chulabhorn Cancer Center, Chulabhorn Royal Academy, Bangkok, Thailand (a tertiary care medical center) from March 2019 to August 2020.

The study methodology was performed and reported in accordance with CONSORT guidelines. No changes were made to the methodology of the study or the trial outcomes after trial commencement. The random allocation was generated by the study statistician, and the methodology to generate the random allocation sequence was computer-generated. Patients were randomized in a 1 : 2 ratio (intervention: control) with a randomized block design.

### 2.2. Participants

The following inclusion and exclusion criteria were considered during patient enrollment. The inclusion criteria were knee pain, diagnosis of moderate to severe (grades 3-4) OA by an orthopedist, following the Kellgren and Lawrence grading system [13] with poor to good grade, employing the Oxford Knee Score [14] for the assessment of the severity of the patient's OA condition in Chulabhorn Hospital, no contraindication to the use of cabbage leaf compression in terms of allergic reactions, and patient's consent to participate. The exclusion criteria were a history of knee surgery for OA, detection of other lesions on the knee area, a clinical history of cabbage allergy, and development of knee OA secondary to an accident or infection.

All patients received appropriate counseling regarding the study objectives and procedures, possible risks and benefits, and alternatives to participation. All procedures performed as part of this study were carried out at the counseling room in the outpatient department. Informed consent for participation was obtained from each patient.

### 2.3. Treatments/Interventions

**Figure 1:**
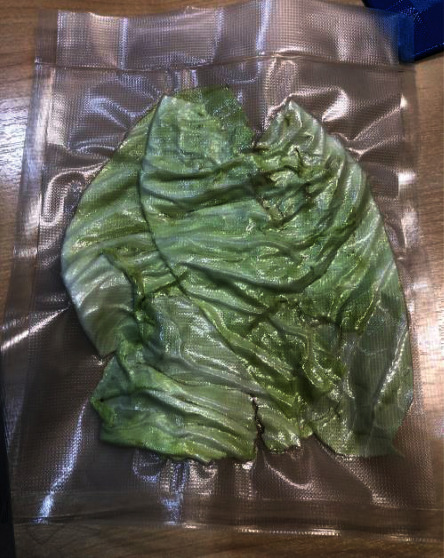
Cabbage leaves in a vacuum-sealed pack.

**Figure 2:**
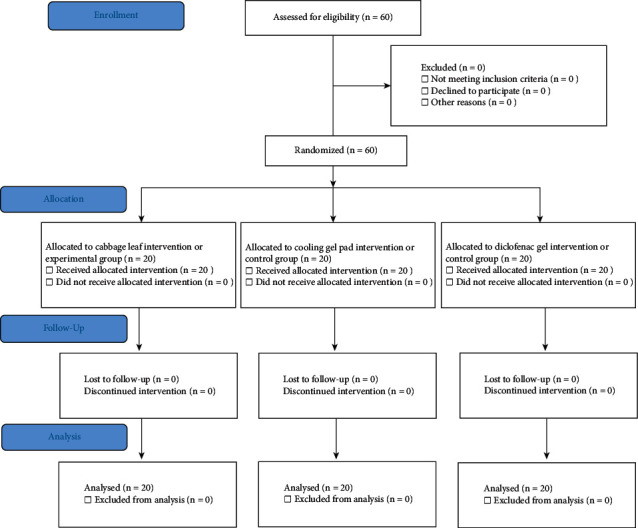
CONSORT flow diagram.

The patient sample was divided into a control group and an experimental group to assess the aspects of osteoarthritic pain relief. The control group comprised 40 patients and was divided into two subgroups of 20 patients each: patients who were treated with reusable cooling gel pads (Nexcare; 3M, Saint Paul, MN, USA) for 30 minutes once a day after dinner or before bedtime by compression on the knee and patients who were treated with 2–4 g of diclofenac gel (diclofenac diethyl ammonium 11.6 mg/g) application rub-in gently four times a day for knee pain relief. The experimental group comprised 20 patients treated with cabbage leaf wrap on the knee, taking 2-3 raw cabbage leaves from the vacuum-sealed pack (Figure 1). This study follows the CONSORT flow diagram (Figure 2).

#### 2.3.1. Cabbage Specifications

Common white cabbage (*Brassica oleracea* L. var. *Capitata* L.) was used in this study. Cabbages were obtained from Golden Acre cabbage plantations in Mae Taeng, Chiang Mai, in the northern region of Thailand. Each cabbage contained at least 20 to 40 leaves, weighed 0.8–2.0 kg, and was harvested after 60 days. Each cabbage leaf weighed 20–50 g, and its size was comparable with that of a person's palm.

#### 2.3.2. Cabbage Leaf Creation

The outer damaged layers of the cabbage leaves were removed from the whole cabbage and the hard stem at the midline cabbage leaf was cut out. The cabbage was then rinsed thoroughly with fresh water and dried properly. Each leafy layer of the cabbage was gently obtained. Each cabbage leaf was individually sealed in a vacuumed plastic bag, which was stored in a standard refrigerator at 3°C for 1 week before therapeutic application. Because of cabbage quality control, selection, and preservation measures, each patient received a fresh cabbage leaf bag (100 grams) that contained 2-3 cabbage leaves in a vacuum-sealed pack every week, and the patient must bruise the leaves using a bottle before applying it to the knee.

### 2.4. Outcomes and Measures

#### 2.4.1. Research Tools and Data Collection Tools

The following methods were employed to collect data from the study patients for the assessment of the efficacy of treatments for pain relief: the Oxford Knee Score for OA severity assessment and the Numerical Rating Scale (NRS) score for pain assessment.

The Oxford Knee Score (OKS) is a self-completed patient-based outcome score. The OKS was developed to specifically assess the patient's perspective of the outcome following total knee arthroplasty. This has subsequently been validated for use in assessing other nonsurgical therapies applied to those suffering from issues with the knee [15]. The OKS consists of twelve questions measuring the function and pain associated with the knee. The questionnaire is short, practical, reliable, valid, and sensitive to clinically important changes over time with 0 being the worst score and 48 being the best score. Scores of 0–19 are perceived as “poor,” 20–29 are perceived as “moderate,” 30–40 are perceived as “good,” and 40–48 are perceived as “excellent” [16]. The Numerical Rating Scale (NRS) score is the measurement of pain intensity in which a respondent selects a whole number (0–10 integers). The NRS is a segmented numeric version of the Visual Analog Scale (VAS) [17]. No changes to the trial outcomes were implemented after the commencement of the trial.

#### 2.4.2. Assessment

The patients assigned to the experimental group were trained to perform the treatment strategy. Each patient was advised to apply the cabbage leaf for 1 hour every day, preferably after dinner or before sleeping. Each patient was advised to assess his or her pain intensity with the Oxford Knee Score before and after cabbage leaf application by utilizing an NRS. All patients of the control and experimental groups underwent a weekly follow-up to pick up vacuum-sealed cabbage leaves. At four weeks, the patients were assessing their pain using the NRS and clinical scores of OA knee with the Oxford Knee Score. If a patient undergoing cabbage leaf application showed worsening pain symptoms, the doctor advised standard treatment instead of cabbage leaf application [18]. If a patient was found to be allergic to the cabbage leaves, the doctor advised immediate cessation of the cabbage leaf application and medical attention at the nearest hospital as soon as possible. The patients were advised to watch for the following signs of allergy during cabbage leaf application: itchiness, urticaria, nausea, vomiting, diarrhea, acute dyspnea, and palpitations. All the patients completed the study successfully, and none of the patients were lost during the follow-up period (Figure 2). The patients were given the contact details of the research team and orthopedist team at Chulabhorn Hospital for referral in case of an emergency.

Medical professionals not involved in the clinical study design and methodology of treatment were responsible for patient screening and recruitment. Each patient assessment report was obtained in a sealed envelope covered by appropriate labeling blinding numbers and delivered to the study statistician for analysis.

#### 2.4.3. Sample Size

For the primary outcome, an average pain intensity of cabbage leaf wraps on the Visual Analog Scale (VAS) of 37.0 ± 23.1 mm at the baseline was expected [7]. Therefore, the minimum calculated sample size was 20 in the experimental group (cabbage leaf intervention), considering a 5% loss to follow-up proportion. The minimal clinically important difference in the Visual Analog Scale pain score in a previous study was 8.6 mm [7]. To evaluate the difference between the Oxford Knee Score and NRS score among interventions and the control group (patients receiving diclofenac gel), the minimal clinically important difference formula was used with an estimated group difference between interventions and the control group at week four with 8.6 ± 8.2 mm, 95% confidence level, and considering 5% loss to follow-up proportion. Therefore, the minimum calculated sample size was 60. The participants were free to withdraw at any time.

### 2.5. Randomization

#### 2.5.1. Sequence Generation

The sequential order was computer generated (Random Allocation Software, version 1.0.0). Nonstratified block randomization with varying block sizes was used to allocate the patients into three groups.

#### 2.5.2. Allocation Concealment Mechanism

The trial coordinators who were responsible for preparing the sealed opaque envelopes at the research site were not involved in the patients' outcome assessments. The opaque envelopes were labeled based on the patients' identification number, and the envelope was opened by the study physician to determine the intervention in ascending order.

### 2.6. Implementation

The trial coordinators generated the random allocation sequence and enrolled the patients in each group. The physician then assigned the patients to the interventions.

### 2.7. Blinding

Neither the patients nor the physician was blinded to the intervention. The outcome assessor was also not blinded since the patients self-reported their outcome assessment.

### 2.8. Statistical Analysis

All analyses were carried out based on the intention to treat the population. Baseline data comparability was ensured by employing analysis of variance (ANOVA) for continuous data and the chi-square test for categorical data. In each group, the average differences in the pain assessment score and Oxford Knee Score before and after the interventions were analyzed by conducting the paired *t*-test with a significance value of 0.05. The differences in the pain score and Oxford Knee Score before and after the intervention of the three study procedures were statistically analyzed by ANOVA. Multiple comparisons of means (Dunnett's test) were performed when the conditions of normality were observed (Kolmogorov–Smirnov test) at a 5% level of significance by employing the STATA version 14.0 (StataCorp, College Station, TX, USA).

## 3. Results

### 3.1. Baseline Demographics and Clinical Characteristics

At baseline, there were no significant differences in the general characteristics of patients with OA, as shown in Table 1. Among the 60 study participants, the cooling gel pad group comprised 3 men and 17 women; the diclofenac gel group comprised 5 men and 15 women; and the cabbage leaf group comprised 2 men and 18 women. There were no significant differences in the sex of patients with OA (*p* value = 0.432). The patients' mean age was 63.83 ± 7.46 years. There were no significant differences in the age of patients with OA (*p* value = 0.194). Forty-three (71.67%) patients had grade 3 OA of the knee (Kellgren and Lawrence grading system). There were no significant differences in osteoarthritis severity of patients with OA (*p* value = 0.720).

### 3.2. Efficacy Outcomes of Interventions

#### 3.2.1. Oxford Knee Score

The mean Oxford Knee Score in the cabbage leaf group was 29.45 ± 6.22 and 33.85 ± 7.28 before and after cabbage leaf application, respectively. The mean Oxford Knee Score in the cooling gel pad group was 25.1 ± 5.72 and 29.6 ± 6.71 before and after cooling gel application, respectively. Finally, the mean Oxford Knee Score in the diclofenac group was 28.45 ± 4.62 and 29 ± 5.39 before and after diclofenac application, respectively (Table 1). There were no significant differences in the Oxford Knee Score among the three groups before (*p* value = 0.185) and after the intervention (*p* value = 0.886).

#### 3.2.2. NRS Score

The mean NRS score in the cabbage leaf group was 4.3 ± 1.72 and 2.15 ± 1.27 before and after cabbage leaf application, respectively. The mean NRS score in the cooling gel group was 5.9 ± 1.17 and 3.8 ± 1.51 before and after cooling gel application, respectively. Finally, the mean NRS score in the diclofenac group was 4.45 ± 1.13 and 4.1 ± 1.71 before and after diclofenac application, respectively (Table 1). There were no significant differences in the NRS Score among the three groups before (*p* value = 0.640) and after the intervention (*p* value = 0.014).

The Oxford Knee Score and NRS score demonstrated clinical improvement in the cabbage leaf group and cooling gel pad group (*p* < 0.001 in both groups, as calculated by paired *t*-test). Hence, the scores in the cabbage leaf and cooling gel pad groups indicated a significant improvement in OA symptoms (*p* < 0.001). In the diclofenac group, however, the Oxford Knee Score (*p*=0.631) and NRS score (*p*=0.413) showed similar improvements. Both scores showed a significance of >0.05 as calculated by the paired *t*-test. These findings indicated that diclofenac gel application did not significantly improve OA symptoms, as shown in Table 2.

#### 3.2.3. Comparisons between the Oxford Knee Score and NRS for Each Intervention

**Figure 3:**
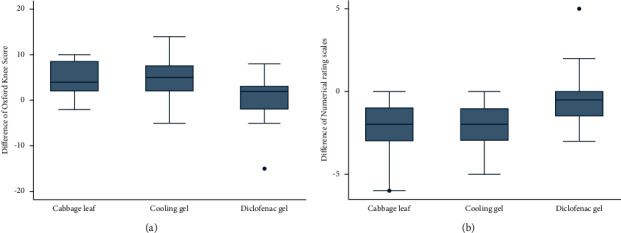
(a) Boxplots showing differences in Oxford knee score and (b) boxplots showing differences in NRS scores.

The overall analysis of the difference between the Oxford Knee Score and the NRS score in the three treatment procedures by ANOVA exhibited a significance value of 0.05. A significant improvement of the Oxford Knee Score (*p* < 0.05) was observed. The differences between the Oxford Knee Score before and after the intervention in the three study procedures were statistically significant (*p*=0.012). Cabbage leaf and cooling gel pad application showed no difference in their performance for improving knee pain (difference, −0.10; 95% CI, −3.57–3.38; *p*=1) (Table 3). However, both groups showed a statistically significant improvement over diclofenac gel (difference, 3.85; 95% CI, 0.30–7.39; *p*=0.03 and difference, 3.95; 95% CI, 0.05–7.85; *p*=0.047, respectively). In addition, more improvement in the Oxford Knee Score was noted in the cabbage leaf and cooling gel application groups than in the diclofenac gel group (Figure 3(a)).

A significant improvement of the NRS score was noted. The difference in the NRS score before and after the intervention was statistically significant in all three groups (*p* < 0.001). Cabbage leaf application and cooling gel pad application exhibited no difference in their performance for reducing knee pain (difference, −0.05; 95% CI, −1.14–1.04; *p*=1) (Table 3). However, both groups showed a statistically significant improvement over the diclofenac gel application group (difference,−1.80; 95% CI, −3.10–−0.50; *p*=0.004 and difference, −1.75; 95% CI, −3.05–−0.45; *p*=0.005, respectively). Moreover, similar improvements in the NRS score of patients using the cabbage leaf and cooling gel pad were observed, but a greater difference was noted than in the diclofenac gel group as shown in Figure 3(b).

## 4. Discussion

### 4.1. Interpretation and Comparison with Other Studies

In this study, cabbage leaf and cooling gel pad application significantly improved knee function and pain compared with diclofenac gel for knee osteoarthritis patients. In addition, complementary and alternative medicine (CAM) provides complementary treatment to relieve chronic disease (including OA) symptoms. The result is consistent with the benefit of CAM with previous studies. For instance, Hashemi et al. [19] reported pomegranate seed powder to have beneficial effects on fasting blood glucose and HbA1c in patients with type 2 diabetes mellitus. Afrasiabian et al. [20] showed that lemon verbena can be suggested as a complementary treatment for patients with insomnia. Zhang et al. [21] demonstrated that acupuncture analgesia is effective in reducing pain. In Thailand, *Derris scandens* has been widely used to reduce musculoskeletal pain [22].

Another CAM, cold application, has been proven to have prominent physiologic effects including vasoconstriction and decreased metabolic activity in patients with musculoskeletal indications, resulting in a decrease of local blood flow, control of swelling, and pain reduction. This leads to a substantial improvement in the limitations of motion and function. Vasoconstriction is known to occur during cold application. After prolonged (>20 minutes) application of cold, the “hunting reaction” may occur at the time of vasodilation as a consequence of the homeostatic mechanism for regulation of temperature. This reaction may result in pain recurrence in the treated joint [23]. The current clinical trial proved that cabbage leaf application is an effective treatment in terms of relieving pain and reducing the severity of OA much more effectively than topical pain medication. Earlier clinical studies carried out by Rath [24] demonstrated the efficacy of cabbage leaf application for the reduction of the pain quotient in patients with OA. Various clinical studies have evaluated the efficacy of alternative topical OA therapies, including herbal treatments such as curcumin ointment [25], *Pistacia atlantica* gum [26], mud bath therapies [27], and traditional herbal formula Xianlinggubao [28] all of which showed considerable improvements in alleviating OA symptoms and decreasing the pain quotient. However, our cabbage leaf therapy demonstrated substantial pain reduction in patients with knee OA with no side effects. Furthermore, our cabbage leaf therapy is an easily accessible and safe option with a low economic burden in terms of hospital expenses for patients with OA undergoing costly treatments with substantial clinically associated side effects and adverse events. Cabbage leaf application has been found to be clinically effective in alleviating the pain of breast congestion in women [8–10]. However, a definitive consensus was not reached in studies of breast congestion and engorgement [29].

In contrast, a definite consensus was reached in the current clinical trial, and the patients who participated were satisfied. The strengths of this study include its randomized study design for balanced outcome observations with little bias and the use of different comparators without the loss of the patients to follow-up. None of the patients were excluded from the study, and no severe adverse effects or progression of OA was observed. However, because the NRS score [30–32] and Oxford Knee Score [14] are subjective evaluations, the reporting strategies may contain bias. Due to the nature of the intervention, complete blinding of the patients was impossible. Therefore, the patients' expectations in the cabbage leaf arm might have interfered with the results, giving a better outcome. Thus, the results observed in the current study might not be strong enough to replace conventional treatment approaches. In this study, we do not allow the patients to prepare the cabbage leaf by themselves at home because the quality and quantity of cabbage leaves for applying to the knee will differ and may affect the study results. Cabbage leaf application can be considered a complement to standard treatment regimens or, in some cases (such as in patients with contraindications to treatment with nonsteroidal anti-inflammatory drugs), alternative treatment approaches with close follow-up can be designed.

### 4.2. Limitations

The main limitation of this study was the lack of blinding the patients and doctors. Additionally, patients with only moderate pain were selected. Therefore, the results cannot be applied to patients with more severe OA. Because of the short duration of the intervention, the progression of OA might not be stopped using these approaches. Obtaining optimal cabbage leaves can be challenging based on patients' geographical localizations. In this study, the most widely available variety of cabbage was selected. Because of the elaborate preparation methods and preservation of the finished product, patients need to visit the hospital weekly to receive a fresh cabbage package; this might pose a significant hindrance to the standard and routine use of this treatment strategy in the future.

### 4.3. Generalizability

Improvements in further clinical studies are needed in terms of preparation strategies and material selection methods. Patient selection criteria can include severe disease, such as that in patients with impending arthroplasty; candidates waiting for the standard operation can also be included to explore the ability to improve their quality of life in the meantime. Long-term clinical studies with a 5-year duration may help to observe OA progression and patient dropout or conversion to arthroplasty with or without cabbage leaf intervention.

## 5. Conclusion

The current study was performed to explore and further the possibility of using cabbage leaves to treat knee pain in patients with OA. The Oxford Knee Score and NRS score were employed to assess the severity and symptoms of OA. The patients were assessed before and after the application of a cabbage leaf, cooling gel pad, and diclofenac gel. The results demonstrated that cabbage leaf and cooling gel pad application significantly improved OA in comparison with diclofenac gel application. Comparison among the clinical procedures proved that the cabbage leaf and cooling gel pad showed similar improvements with statistical significance and greater efficacy than diclofenac gel application. Although it is more convenient to use a cooling gel pad to treat OA knee, it is more beneficial to use cabbage leaf in some areas, especially rural areas where it is an inexpensive home remedy to relieve knee pain.

## Figures and Tables

**Table 1:** General baseline characteristics of patients with OA.

Variables	Cabbage leaf (*N* = 20)	Cooling gel (*N* = 20)	Diclofenac gel (*N* = 20)	Total	*p* value
Sex					
Male	2 (10)	3 (15)	5 (25)	10 (16.67)	0.432
Female	18 (90)	17 (85)	15 (75)	50 (83.33)	

Age (years)	63.85 ± 7.17	61.80 ± 8.48	65.85 ± 6.42	63.83 ± 7.46	0.194

Osteoarthritis severity (Kellgren and Lawrence grading system)					
3	15 (75)	15 (75)	13 (65)	43 (71.67)	0.720
4	5 (25)	5 (25)	7 (35)	17 (28.33)	

Oxford knee score (points)					
Before	29.45 ± 6.22	25.1 ± 5.72	28.45 ± 4.62	27.67 ± 5.78	0.185
After	33.85 ± 7.28	29.6 ± 6.71	29 ± 5.39	30.82 ± 6.76	0.886

NRS score (points)					
Before	4.3 ± 1.72	5.9 ± 1.17	4.45 ± 1.36	4.88 ± 1.58	0.640
After	2.15 ± 1.27	3.8 ± 1.51	4.1 ± 1.71	3.35 ± 1.72	0.014^*∗*^

Data are presented as *n* (%) or mean ± standard deviation. NRS: numerical rating scale. ^*∗*^Significant at 0.05.

**Table 2:** Comparison of the mean difference between the three groups by using the paired *t*-test.

Assessment	Group	Mean difference (SD)	95% CI of the difference		Paired *t*-test *p* value
			Lower	Upper	
Oxford Knee Score (before-after)	Cabbage leaf	4.4 (3.87)	2.59	6.21	<0.001^*∗*^
	Cooling gel	4.5 (4.87)	2.22	6.78	<0.001^*∗*^
	Diclofenac gel	0.55 (5.03)	−1.80	2.90	0.631

NRS score (before-after)	Cabbage leaf	−2.15 (1.38)	−2.80	−1.50	<0.001^*∗*^
	Cooling gel	−2.10 (1.37)	−2.74	−1.46	<0.001^*∗*^
	Diclofenac gel	−0.35 (1.87)	−1.23	0.53	0.413

NRS: Numerical Rating Scale; SD: standard deviation; CI: confidence interval. ^*∗*^Significant at 0.05.

**Table 3:** Multiple comparisons of differences among procedures by using Dunnett's test.

Assessment	Group	Group	Mean difference (SE)	95% CI of difference		Dunnett's test *p* value
				Lower	Upper	
Oxford knee score	CL	CG	−0.1 (1.39)	−3.57	3.38	1
		DG	3.85 (1.42)	0.30	7.39	0.03^*∗*^
	CG	DG	3.95 (1.57)	0.05	7.85	0.047^*∗*^

NRS score	CL	CG	−0.05 (0.44)	−1.14	1.04	1
		DG	−1.80 (0.52)	−3.10	−0.50	0.004^*∗*^
	CG	DG	−1.75 (0.52)	−3.05	−0.45	0.005^*∗*^

CL: cabbage leaf; CG: cooling gel pad; DG: diclofenac gel. NRS: numerical rating scale; SE: standard error; CI: confidence interval. ^*∗*^Significant at 0.05.

## Data Availability

The data supporting this study's findings are available from the corresponding author upon reasonable request.
